# Dysbiosis, gut barrier dysfunction and inflammation in dementia: a pilot study

**DOI:** 10.1186/s12877-020-01644-2

**Published:** 2020-07-20

**Authors:** Vanessa Stadlbauer, Lara Engertsberger, Irina Komarova, Nicole Feldbacher, Bettina Leber, Gerald Pichler, Nicole Fink, Monika Scarpatetti, Walter Schippinger, Reinhold Schmidt, Angela Horvath

**Affiliations:** 1 Department of Internal Medicine, Division of Gastroenterology and Hepatology, Medical University of Graz, Auenbruggerplatz 15, 8036 Graz, Austria; 2 Center of Biomarker Research in Medicine (CBmed), Graz, Austria; 3 Department of Surgery, Division of Transplantation Surgery, Medical University of Graz, Graz, Austria; 4 Department of Neurology, Geriatric Health Centers Graz, Albert Schweitzer Hospital, Graz, Austria; 5 Clinical Division of Neurogeriatrics, Department of Neurology, Medical University of Graz, Graz, Austria

**Keywords:** Microbiome, Diversity, Gut barrier, Inflammation, Cognitive function, Butyrate producer

## Abstract

**Background:**

Dementia is an increasing public health threat worldwide. The pathogenesis of dementia has not been fully elucidated yet. Inflammatory processes are hypothesized to play an important role as a driver for cognitive decline but the origin of inflammation is not clear. We hypothesize that disturbances in gut microbiome composition, gut barrier dysfunction, bacterial translocation and resulting inflammation are associated with cognitive dysfunction in dementia.

**Methods:**

To test this hypothesis, a cohort of 23 patients with dementia and 18 age and sex matched controls without cognitive impairments were studied. Gut microbiome composition, gut barrier dysfunction, bacterial translocation and inflammation were assessed from stool and serum samples. Malnutrition was assessed by Mini Nutritional Assessment Short Form (MNA-SF), detailed information on drug use was collected. Microbiome composition was assessed by 16S rRNA sequencing, QIIME 2 and Calypso 7.14 tools.

**Results:**

Dementia was associated with dysbiosis characterized by differences in beta diversity and changes in taxonomic composition. Gut permeability was increased as evidenced by increased serum diamine oxidase (DAO) levels and systemic inflammation was confirmed by increased soluble cluster of differentiation 14 levels (sCD14). BMI and statin use had the strongest impact on microbiome composition.

**Conclusion:**

Dementia is associated with changes in gut microbiome composition and increased biomarkers of gut permeability and inflammation. *Lachnospiraceae NK4A136 group* as potential butyrate producer was reduced in dementia. Malnutrition and drug intake were factors, that impact on microbiome composition. Increasing butyrate producing bacteria and targeting malnutrition may be promising therapeutic targets in dementia.

**Trial registration:**

NCT03167983.

## Background

Dementia leads to disability and dependency among older people worldwide and thereby has enormous physical, psychological, social and economic impact on patients, caregivers, families and society [1]. Alzheimer’s disease (AD) is the most common form of dementia accounting for 60–70% of the cases [1]. In AD, pathologic protein aggregates and neuroinflammation mediated by microglia cells are involved in the pathogenesis, however, the exact mechanism is still unclear [2]. Microglia maturation and function is critically dependent on short-chain fatty acids produced by the gut microbiome and therefore highlights the microbiome as a potential diagnostic and therapeutic target in dementia [3]. During ageing, the gut microbiome decreases in diversity, loses beneficial taxa and facultative pathogens increase [4]. Diet and the place of residence play an important role in the shaping of the microbiome [5, 6]. Ageing is also associated with inflammation – often termed as “inflammageing”. Inflammation is further associated with an increase in gut permeability, mucosal inflammation and bacterial translocation [2].

Since the main risk factor for developing dementia, especially AD, is ageing, it can be hypothesized that the gut-brain axis is a possible link between age and disease related dysbiosis and inflammation. Animal studies suggest that AD is associated with changes in the gut microbiome composition with a decrease in beneficial, anti-inflammatory genera [7]. Furthermore, genetic alterations in amyloid genes can influence microbiome composition in mice, pointing towards a vicious cycle in AD development [8]. Recently studies from the USA have identified a loss in species diversity and differences in bacterial composition in the stool of AD patients compared to matched controls [9]. A study from Japan has also shown that gut microbiome composition is independently and strongly associated with dementia [10]. Furthermore, it has been recently published that the microbiome of dementia patients causes a dysregulation of the anti-inflammatory P-glycoprotein pathway [11].

So far factors that may influence the composition of the gut microbiome in patients with dementia have not been studied in detail. Potentially influencing factors could be malnutrition, which is common in dementia and associated with disease severity, [12–14] or drug intake, since polypharmacy is a common problem in elderly persons and the impact of drugs on the microbiome has recently gained attention [15–17].

We hypothesize that dementia is associated with dysbiosis, gut barrier dysfunction and inflammation and we aim to identify external factors influencing microbiome composition in dementia, such as nutrition and drug intake. To study this, we conducted a prospective controlled cohort study in patients with dementia and age matched controls.

## Methods

Between July 2017 and March 2018 we recruited 25 patients with diagnosis of Alzheimer type (*n* = 21) or mixed type (Alzheimer type plus vascular type, *n* = 4) dementia with a Mini Mental State Examination (MMSE) ≤ 26 and 18 age and sex matched controls without evidence of dementia and a MMSE > 26 at the Albert-Schweitzer Hospital Graz and at the University Hospital Graz. Diagnosis of dementia was made by a board-certified neurologist/psychiatrist and according to ICD10 criteria including cerebral imaging and exclusion of differential diagnosis by full laboratory workup. Participants *or* their legal representative gave written informed consent. We excluded participants with other forms of dementia, inflammatory bowel diseases, liver cirrhosis or recent (< 4 weeks) antibiotic or probiotic treatment. The study (29–420 ex 16/17) was approved by the ethics committee Ethic Committee of the Medical University of Graz (IRB00002556) and has been registered at clinicaltrials.gov (NCT03167983) before the study started. The study was performed according to the Declaration of Helsinki and Good Clinical Practise guidelines. Written informed consent was obtained before any study specific procedure was performed from participants or their legal representatives (in case patients were not able to give written consent any more due to the severity of cognitive dysfunction). Routine blood biochemistry analysis including full blood count, electrolytes, renal function, liver function, albumin and total protein levels and inflammation parameter and a detailed medical history was performed. Stool and serum samples were collected for analysis of gut microbiome composition and biomarkers of intestinal permeability, inflammation and bacterial translocation. Serum samples were collected after overnight fasting. Stool samples were collected by the patients or caregivers in sterile collection tubes either on the same day or the evening before the study visit. Samples were kept on 4 °C until arrival at the hospital and then frozen immediately at − 80 °C. Mini Nutritional Assessment Short Form [18] was used to assess nutritional status.

### Cognitive function

The Mini-Mental State Examination [19] and the clock drawing test [20] were used to quantify cognitive function. We classified cognitive dysfunction according to the German S3-guideline on Dementia 2016 as MMSE 0–9: severe; MMSE 10–19: moderate; MMSE 20–26: mild; MMSE 27–30: no dementia [21].

### Microbiome analysis

Total DNA was isolated from frozen stool samples using MagnaPure LC DNA Isolation Kit III (Bacteria, Fungi) (Roche, Mannheim, Germany) according to manufacturer’s instructions including mechanic and enzymatic lysis [22]. Hypervariable regions V1-V2 were amplified in a target specific PCR using the primers 27F and R357 (27F-AGAGTTTGATCCTGGCTCAG; R357-CTGCTGCCTYCCGTA) and sequenced with the Illumina MiSeq technique (Illumina, Eindhoven, The Netherlands) [22]. Sequencing was done in cooperation with the Core Facility for Molecular Biology at the Center for Medical Research in Graz.

### Gut permeability, inflammation and bacterial translocation

Enzyme linked immunosorbent assays (ELISA) were used to measure fecal and serum calprotectin, fecal and serum zonulin, serum diamine oxidase (Immundiagnostic AG, Bensheim, Germany), soluble (s)CD14 (R&D Systems, Minneapolis, USA), and lipopolysaccharide binding protein (LBP) (Hycult biotech, Uden, The Netherlands). All assays were performed according to manufacturers’ instructions. Bacterial products (endotoxin, peptidoglycan and bacterial DNA) were detected in serum using HEK-Blue hTLR4, HEK-Blue hNOD2 and HEK-Blue hTLR9 reporter cells (Invivogen, Toulouse, France), respectively as published previously [23].

### Statistical analysis

For microbiome analysis generated FASTQ files were processed for analysis using Qiime2 [24] tools implemented in Galaxy (https://galaxy.medunigraz.at). Denoising (primers removing, quality filtering, correcting errors in marginal sequences, removing chimeric sequences, removing singletons, joining paired-end reads, and dereplication) was done with DADA2 [25]. Taxonomy was assigned based on Silva 132 database release at 99% OTU level, trained using a Naïve Bayes classifier. Sequences were blasted in the NCBI database for further classification [26]. Features with a total sequence count of less than 10 and/or present in less than two patient samples were excluded from analysis. Chloroplast and cyanobacteria filtering were performed to remove contaminants. The resulting mean sequencing depth was 41,631 (range 21,774–53,719) reads per sample. In QIIME2, “feature” is the observational unit and describes a sequence variant/operational taxonomic unit. Analysis was done using the web-based software Calypso 7.14 (http://cgenome.net/calypso/) [27]. For alpha diversity assessment, data was rarefied with a sampling depth of 24,771 reads and Chao1 index, Simpson reciprocal index and Faith phylogenetic diversity were calculated to quantify microbial diversity.

Beta diversity and taxon comparison was done on an unrarefied feature table after total sum scaling and square root transformation. Redundancy analysis based on Bray Curtis dissimilarity was used to compare beta diversity between groups and to identify significant confounders. Differentially abundant taxa were identified with Analysis of Composition of Microbiomes (ANCOM) [28]. This method accounts for compositional constraints and reduces false discovery rates while maintaining high statistical power during the detection of differentially abundant taxa. This test utilizes multiple taxon-to-taxon comparisons and infers differential abundance of a taxon based on the number of significant group comparisons relative to other taxa (W-value). Feature selection was performed using the supervised machine learning tool Linear Discriminant analysis Effect Size (LEfSe) [29]. LEfSe is a tool to discover features by way of class comparison, tests of biological consistency and effect size estimation between two or more microbial communities. All analyses were performed on feature, genus, family, class, order and phylum level. Sequence data is publicly available at the NCBI Sequence Read Archive (SRA accession: PRJNA608281).

All other statistical analyses were performed using SPSS version 25.0 (SPSS Inc., Chicago, Illinois, USA) and R [30] version 3.5.2 (packages: “mice”, “ggcorrplot”, “psych”, “randomForest”, “fmsb”, “stats”, “robustHD”) [31–37]. Tests (t-test or Mann-Whitney) were chosen depending on the distribution of the data assessed by Shapiro-Wilk normality test. Spearman rank correlation with Benjamini-Hochberg correction for multiple testing was used to assess strength and direction of linear relationships between variables. All statistical tests were 2-sided, and *p*-values < 0.05 were considered statistically significant. Data are presented as median and 95% confidence interval unless stated otherwise. Missing values were imputed by multivariate imputation by chained equations (package “mice”) [33] based on random forests (package “randomForest”) [31]. Univariate and multivariate RDA was performed to find out which variables explain the variance in microbiome composition. VIF values were calculated to account for collinearity between the explanatory variables (package “fmsb”), [37] explanatory variables were standardized for multivariate RDA (package “robustHD”) [35]. Network analysis was based on Spearman’s rho associations between taxa and converting the pairwise correlations into dissimilarities to ordinate nodes in a two dimensional PCoA plot.

## Results

### Patient characteristics

**Fig. 1:**
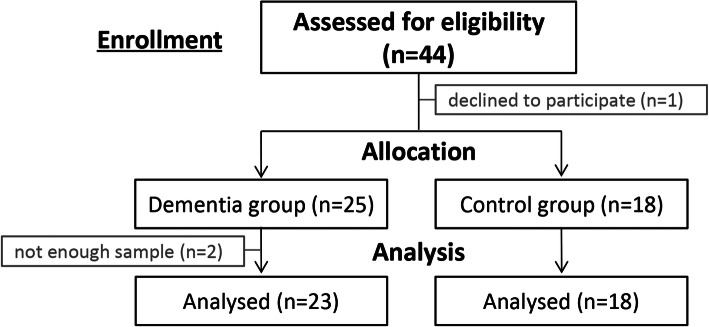
Patient flow chart

We recruited 25 patients with dementia (Alzheimer type and mixed type) and 18 matched controls without cognitive impairment in this prospective controlled cohort study. From 2 dementia patients we were not able to collect enough stool and blood samples to do the intended analyses; therefore, they were excluded from the final analysis. (Fig. 1) Dementia patients had a lower body mass index (BMI) and erythrocyte count as well as lower serum albumin and total protein levels compared to controls. Accordingly, nutritional status according to MNA-SF was significantly worse in dementia patients. Within the dementia group, erythrocyte count (*r* = 0.669, *p* = 0.002) and albumin (*r* = 0.707, *p* < 0.002) showed a significant positive correlation with MMSE and clock drawing test showed a weak positive correlation with albumin (*r* = 0.485, *p* = 0.019). No significant differences were found regarding age, gender, and other routine biochemistry parameters. BMI did not correlate with MMSE or clock drawing test results. Collinearity analysis showed variance inflation factors (VIF) below 2 for MMSE, clock-drawing test, BMI, albumin and MNA-SF.

Prescription drug intake was significantly different between dementia patients and controls. Dementia patients took three times more prescription drugs compared to controls. Antidepressants, laxatives, opioids, anti-dementia drugs, sedatives, vitamin D and metamizole were prescribed nearly exclusively in the dementia group, whereas proton pump inhibitors (PPI), antihypertensive drugs, statins, nonsteroidal anti-inflammatory drugs (NSAIDS), paracetamol, antidiabetics, thyroid hormones, calcium and magnesium supplements, anticoagulation and phytotherapeutics were equally prescribed for dementia patients and age matched controls. Laxatives, sedatives, metamizole and paracetamol were usually prescribed as needed, whereas the other drugs were prescribed as fixed dose medication. Patient characteristics are shown in Table 1.

**Table 1:** Patient characteristics. Data are given as median and 95% confidence interval unless stated otherwise

	Dementia patients (*n* = 23)	Controls (*n* = 18)	*p*-value
Age (years)	88 (73;85)	75 (74;76)	n.s.
Gender (f/m) (n)	15/8	11/7	n.s.
BMI (kg/m2)	24.8 (22.6; 25.9)	28.1 (25.2; 31.0)	*p* = 0.028
MMSE	16 (13;21)	29 (30;30)	*p* < 0.0001
Clock drawing test	3 (0;5)	7 (7;9)	*p* < 0.0001
Number of prescription drugs	9 (6;11)	3 (1;4)	*p* < 0.0001
MNA-SF	10 (9;12)	14 (14;14)	*p* < 0.0001
Leukocytes (10^9^/L)	6.6 (6.2;8.3)	6.1 (5.4;7.5)	n.s
Erythrocytes (10^12^/L)	4.5 (4.0;4.7)	4.7 (4.4; 5.1)	*p* = 0.028
Thrombocytes (10^9^/L)	220 (216;248)	216 (205;222)	n.s
Hemoglobin g/dL	13.2 (12.7;14.4)	14.1 (13.3;14.7)	n.s
Creatinine (mg/dL)	0.9 (0.8;1.0)	1.0 (0.9;1.1)	n.s
Bilirubin (mg/dL)	0.6 (0.5;0.9)	0.6 (0.5;0.6)	n.s
Albumin (g/dL)	3.9 (3.7; 4.1)	4.2 (4.1;4.4)	*p* = 0.006
total protein (g/dL)	7.0 (6.8;7.3)	7.5 (7.3;7.6)	*p* = 0.014
CRP (mg/l)	5 (3;11)	2 (1.2; 3.4)	n.s.

*BMI* body mass index, *MMSE* Mini mental state examination, *MNA-SF* Mini Nutritional Assessment Short Form, *CRP* C reactive protein

### Gut microbiome composition

**Fig. 2:**
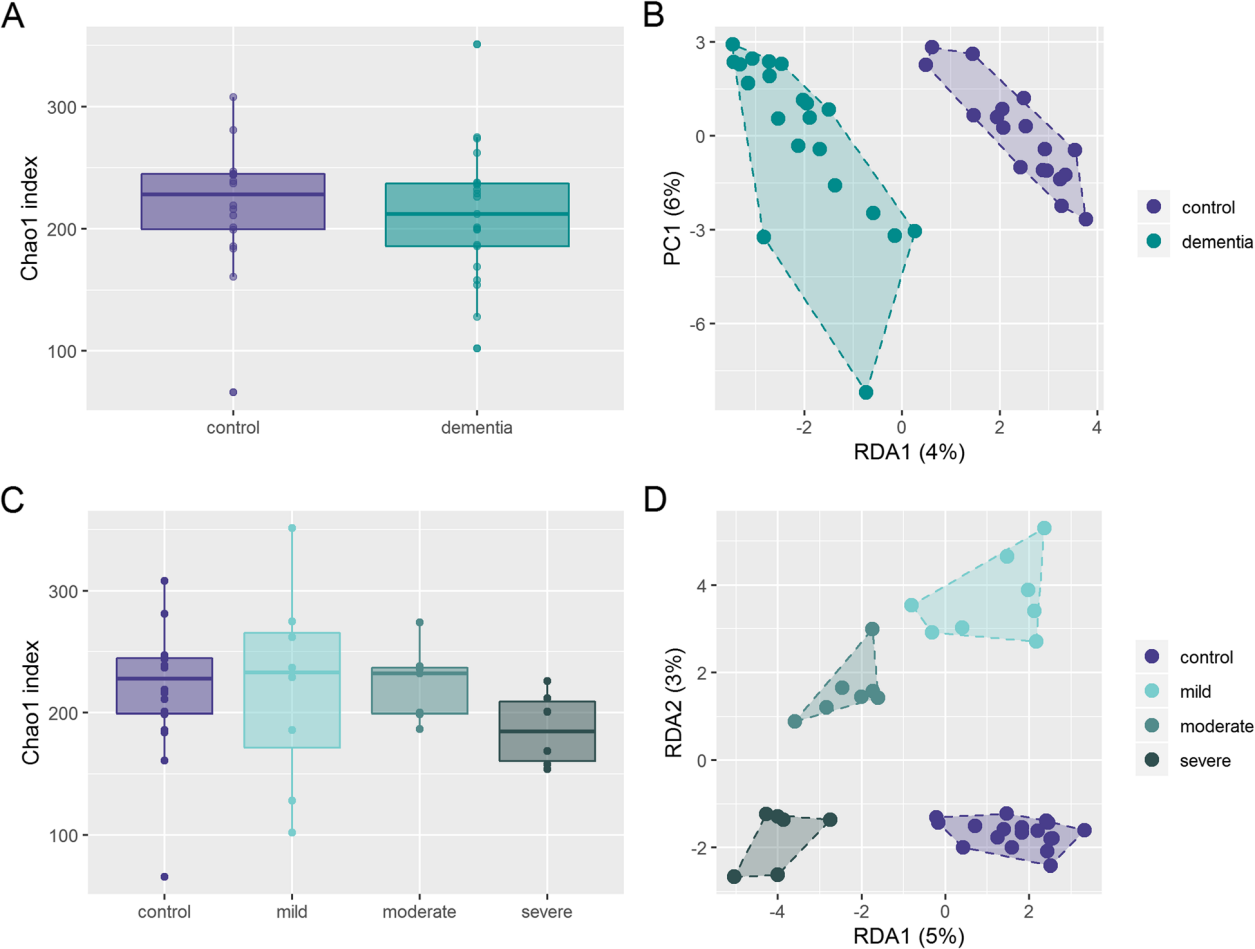
Differences in alpha and beta diversity in stool microbiome between dementia patients and controls **a** Alpha diversity (Chao1) in dementia patients and controls **b** Beta diversity (RDA) in dementia patients and controls **c** Alpha diversity between controls and different stages of cognitive dysfunction **d** Beta diversity (RDA) in controls and different stages of dementia

**Fig. 3:**
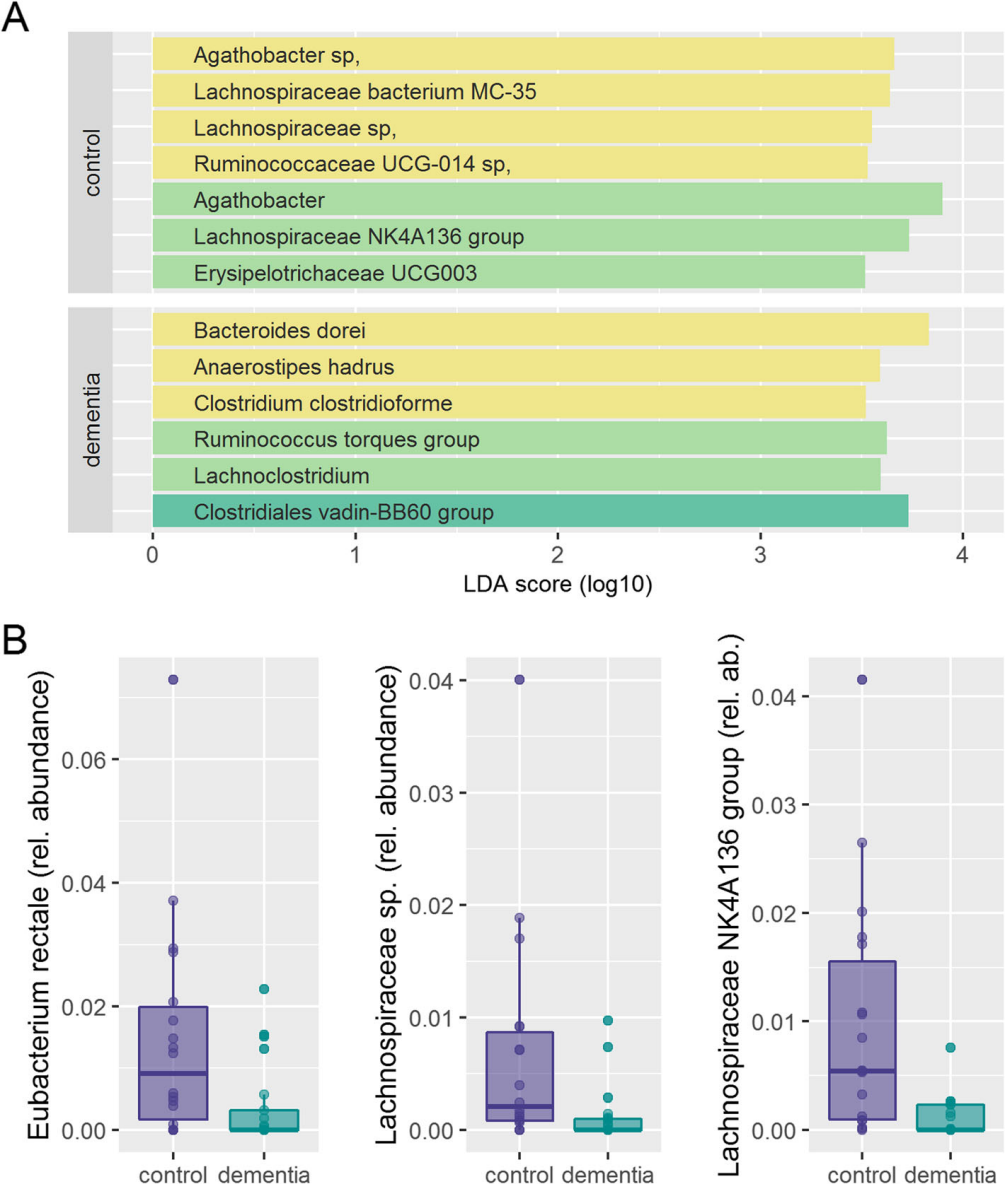
**a** Features selected by Linear discriminant analysis Effect Size (LEfSe) to discriminate between dementia patients and controls. **b** Differentially abundant taxa between dementia and controls

**Fig. 4:**
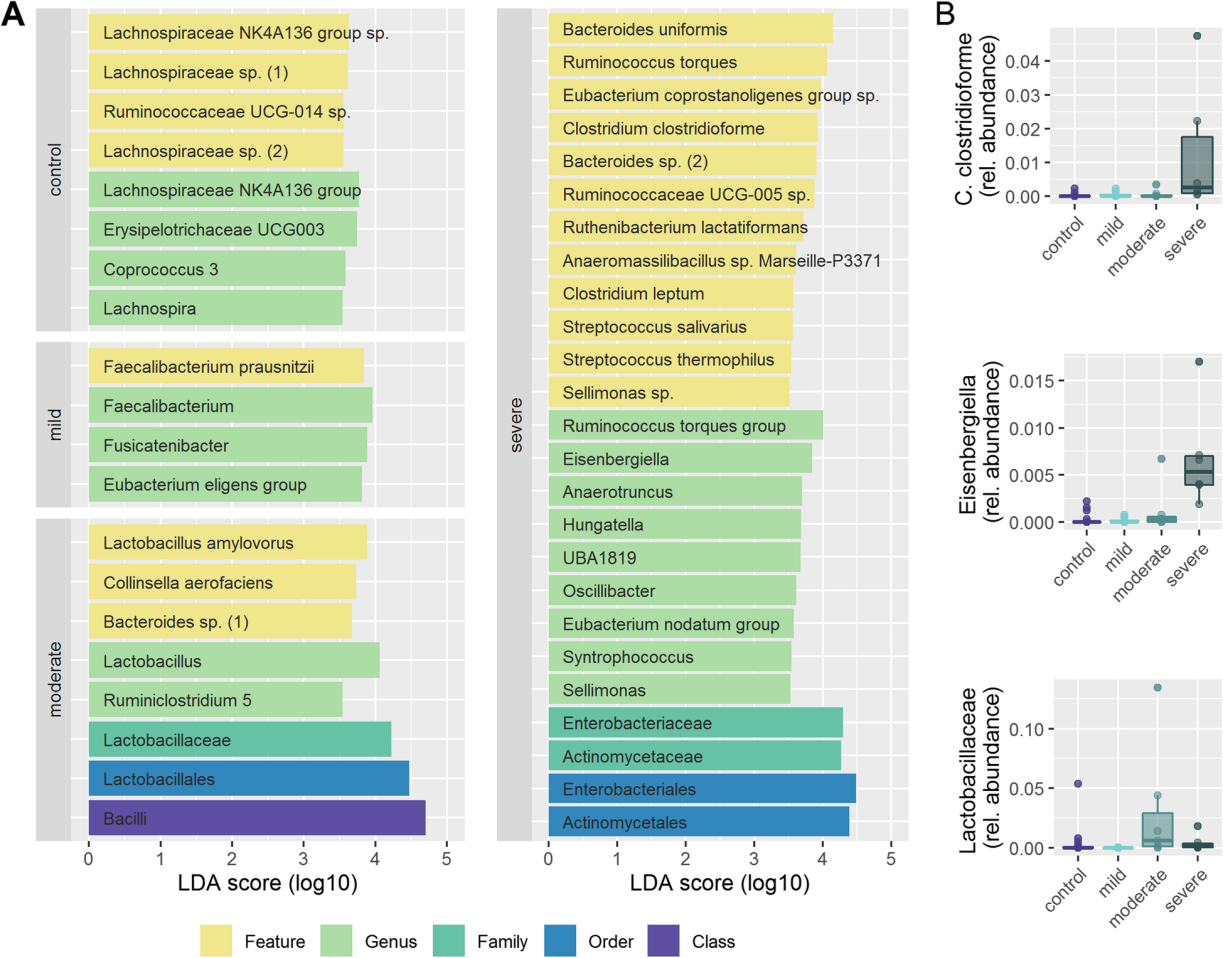
**a** Features selected by Linear discriminant analysis Effect Size (LEfSe) to discriminate between dementia different stages of cognitive dysfunction and controls. **b** Differentially abundant taxa between stages of cognitive dysfunction

Alpha diversity using Chao 1 index (Fig. 2a), Simpson reciprocal index (Supplementary figure S1A) or Faith phylogenetic diversity (Supplementary figure S1C) was not significantly different in dementia patients compared to age matched controls. Redundancy Analysis (RDA) showed clear clustering of dementia patients compared to controls (Variance 34.3, F = 1.31 *p* = 0.003). (Fig. 2b) Alpha diversity also did not change significantly with increasing degree of dementia. (Fig. 2c and supplementary figure S1B and D) RDA showed clear clustering of different stages of dementia (Variance 94.7, F = 1.2 *p* = 0.001). (Fig. 2d) Linear discriminant analysis of effect size (LEfSe) identified one family, 5 genera and 7 features to differ between patients with dementia and controls. For example, the features *Clostridium clostridioforme, Anaerostipes hadrus* and *Bacteroides dorei* were associated with dementia; *Lachnospiraceae bacterium MC-35*, another *Lachnospiraceae sp*., and the genus *Lachnospiraceae NK4A136 group* were associated with health. (Fig. 3a) Analysis of Composition of Microbiomes (ANCOM) confirmed that from the taxa identified by LEfSe to discriminate between dementia and control, one uncultured *Lachnospiraceae* feature as well as the genus *Lachnospiraceae NK4A136 group* were significantly less abundant in stool of dementia patients. Additionally, the feature *Eubacterium rectale* was also less abundant in stool of dementia patients (Fig. 3b). When looking at different stages of dementia, LEfSe identified one class, 3 orders, 3 families, 18 genera and 20 features, being associated with severity of cognitive impairment. Most notably, three *Lachnospiraceae* species with the corresponding genus *Lachnospiraceae NK4A136 group* and the genus *Lachnospira* were associated with health; *Faecalibacterium prausnitzii* was associated with mild dementia; moderate dementia was associated with *Lactobacillus amylovorus* and the corresponding higher taxonomic levels (the genus *Lactobacillus*, the family *Lactobacillaceae* and the order *Lactobacillales*). Severe dementia was associated with several potential pathogens (e.g. *Clostridium clostridiforme, Streptococcus salivarius*) (Fig. 4a) From these discriminating taxa, ANCOM analysis identified the feature *C. clostridioforme* and the genus *Eisenbergiella* to increase with severity of cognitive impairment and the family *Lactobacillaceae* to be highest in patients with moderate cognitive impairment. (Fig. 4b).

### Association of drug intake and nutrition on microbiome composition

While some drugs were nearly exclusively prescribed in dementia patients (for details see supplementary table S1), other drugs were equally prescribed between dementia and control subjects. To understand how drug use may influence microbiome composition irrespective of the disease, we studied the effect of drugs that were equally prescribed in dementia patients and healthy controls on diversity measures and taxonomic composition: namely PPI, antihypertensive drugs, statins, thyroid hormones and NSAIDS. Paracetamol, antidiabetics and calcium and magnesium supplements were taken by less than 15% of the cohort and therefore these drugs were not included into the analysis. None of the drugs influenced alpha diversity (Chao1, Simpson, Faith phylogenetic diversity). Statins, but none of the other drugs had a significant impact on beta diversity (RDA, Variance 35.58, F 1.36, *p* = 0.003). LEfSe identified several features, genera, families, orders and classes being associated with use or non-use of each drug. ANCOM identified several of these taxa as well as some taxa that were not discriminative on LEfSe to be differentially abundant taxa between drug user and non-user. For details see supplementary tables S2–6. Interestingly, PPI use was associated with increased abundance of oral bacteria (e.g. *Streptococcus salivarius*) whereas statin and antihypertensive drug use was associated with increased abundance of bacteria known to produce butyrate (e.g. *Faecalibacterium sp*.).

Since malnutrition was present in 74% of dementia patients but in none of the control persons, microbiome composition in malnourished versus non-malnourished patients was very similar to the results obtained when comparing dementia versus controls. LEfSe identified the feature *Ruminococcaceae UCG-014 sp* with the corresponding genus *Ruminococcaceae UCG014* and the genus *Lachnospiraceae NK4A136 group* to be associated with normal nutritional state. These taxa were also found to be associated with healthy controls. The genus *Eubacterium hallii group* was associated with dementia. (supplementary table S7) ANCOM confirmed the feature *Ruminococcaceae UCG-014 sp*. and the genus *Lachnospiraceae NK4A136 group* to be differentially abundant between malnutrition and normal nutrition.

### Gut barrier dysfunction, inflammation and bacterial translocation

We assessed intestinal permeability by serum diaminooxidase (DAO) and fecal zonulin; inflammation by C-reactive protein, serum lipopolysaccharide binding protein (LBP), soluble CD 14 (sCD14) and fecal calprotectin as well as bacterial translocation by endotoxin, peptidoglycanes and bacterial DNA in serum. Patients with dementia had higher DAO levels and sCD14 levels, indicative for an association with increased gut permeability and increased endotoxin load. (Table 2).

**Table 2:** Biomarker for gut barrier dysfunction, inflammation and bacterial translocation, Data are shown as median and 95% confidence interval

	Dementia patients (*n* = 23)	Controls (*n* = 18)	*p*-value
Serum diaminooxidase (U/ml)	20.8 (9.7;29)	11.2 (8.4; 13.8)	0.025
Fecal zonulin (ng/ml)	33.8 (31.2; 57)	55.1 (40.8; 76.7)	n.s
C-reactive protein (mg/L)	5 (4; 11)	2 (1.2;3.4)	n.s
Serum lipopolysaccharide binding protein (μg/ml)	17.9 (16.1; 18.6)	20.0 (14.6; 21.3)	n.s
Soluble CD 14 (μg/ml)	2.4 (1.9; 3.1)	1.8 (1.7; 2.1)	0.022
Fecal calprotectin (ng/ml)	31.5 (26.6; 85.8)	49.0 (18.2; 66.3)	n.s
Endotoxin (EU/ml)	0.26 (0.0; 0.33)	0.25 (0.09; 0.53)	n.s
Peptidoglycan^a^ (ng/mL)	0.96 (0.26; 1.66)	0.42 (0.30;1.05)	n.s.
Bacterial DNA (μM)	0.06 (0.00;1.46)	0.7 (0.0; 1.29)	n.s

^a^peptidoglycan was only measurable in 12% of the samples, therefore median and confidence interval only for the positive samples are shown. *CD* cluster of differentiation, *EU* endotoxin units

PPI use was associated with significantly increased faecal calprotectin levels (PPI use: 92.5 ng/ml (50.2; 120.5); PPI non-use: 28.1 ng/ml (20.8; 47.9); *p* = 0.008). Antihypertensive use was associated with significantly increased CRP levels (antihypertensive use: 6 mg/dl (3; 11); antihypertensive non-use 1.3 mg/dl (1;4); *p* = 0.016), suggesting complex relations between disease, drug use and inflammation.

### Multivariate and network analysis of potential factors influencing microbiome composition in dementia

**Fig. 5:**
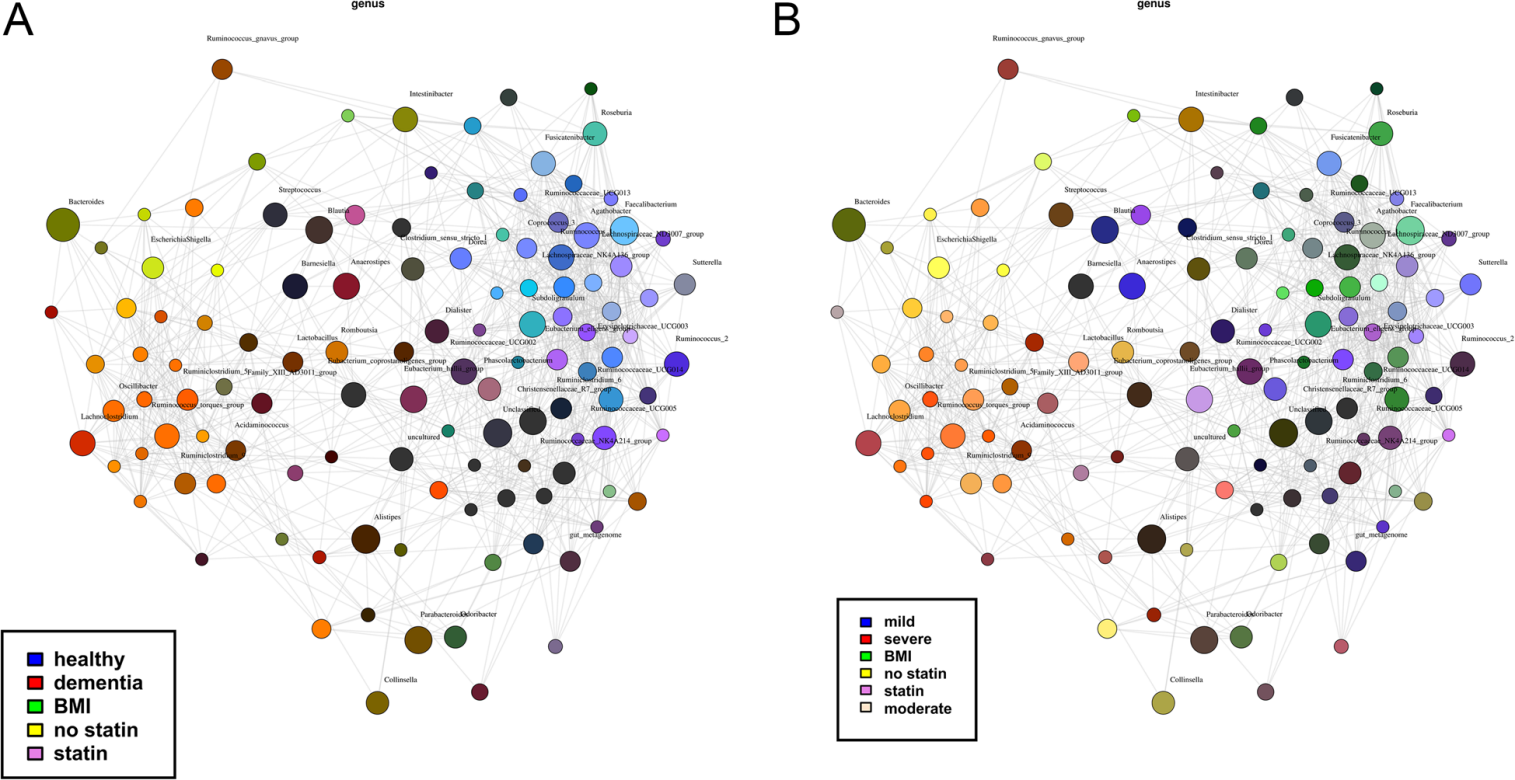
Network analysis to identify associations between bacteria and selected host variables. Taxa and explanatory variables are represented as nodes, taxa abundance as node size, and edges represent positive associations. Nodes (genera) are coloured based on their association with selected host variables (dementia/health, dementia stages, statin use or non-use, BMI). **a** whole cohort (*n* = 41), **b** dementia patients (*n* = 21)

To understand the main drivers of dysbiosis in dementia we further performed univariate and multivariate RDA to assess the association of clinical variables and biomarkers with microbiome composition. RDA showed that BMI, albumin, total protein, sCD14, statins, NSAIDs, number of drugs, MNA-SF, MMSE, clock-drawing test, sex, number of drugs were explanatory variables for microbiome composition in controls compared to dementia and between different stages of cognitive dysfunction (*p* < 0.1) (supplementary table S8). To the final multivariate RDA model explanatory variables with *p* < 0.1 in the univariate analysis were included and variables with VIF > 2 in multicollinearity analysis were excluded. (Table 3) In the multivariate model BMI and statin use were the remaining significant explanatory variables for differences in microbiome composition between dementia and control groups and between the groups of dementia severity in the dementia group only. (Table 3) Network analysis also illustrates the overlap between factors influencing microbiome composition: Genera associated with dementia (red) overlap with genera associated with no statin intake (yellow) and BMI (green), whereas genera associated with health (blue) overlap with genera associated with statin intake (purple). (Fig. 5a) When performing network analysis in the subgroup of dementia patients only, the overlaps are less clear, but again genera associated with severe dementia (red) overlap with genera associated with no statin intake (yellow) and genera associated with mild dementia (blue) overlap with genera associated with statin intake (purple). The association with BMI is less pronounced in the dementia subgroup. (Fig. 5b).

**Table 3:** Multivariate RDA to identify the most important explanatory variables for microbiome composition changes

Variable	Control versus Dementia	Severity of dementia
BMI	Variance = 33.18	Variance = 33.18
	F = 1.29	F = 1.29
	***P*** **= 0.006**	***P*** **= 0.008**
Total protein	Variance = 29.19	Variance = 29.19
	F = 1.14	F = 1.14
	*P* = 0.067	*P* = 0.070
soluble CD14	Variance = 29.06	Variance = 29.06
	F = 1.13	F = 1.13
	*P* = 0.072	*P* = 0.079
Statins	Variance = 32.06	Variance = 32.06
	F = 1.25	F = 1.25
	***P*** **= 0.009**	***P*** **= 0.014**
Clock-drawing test	Variance = 25.89	Variance = 25.89
	F = 1.01	F = 1.01
	*P* = 0.376	*P* = 0.374
Age	Variance = 25.88	Variance = 25.88
	F = 1.01	F = 1.01
	*P* = 0.427	*P* = 0.409
Sex	Variance = 27.63	Variance = 27.63
	F = 1.08	F = 1.08
	*P* = 0.137	*P* = 0.154
NSAIDS	Variance = 28.17	Variance = 28.17
	F = 1.10	F = 1.10
	*P* = 0.098	*P* = 0.107

*BMI* body mass index, *NSAIDS* non-steroidal anti-inflammatory drugs

## Discussion

Our cross-sectional controlled pilot cohort study shows that dementia is associated with changes in microbiome composition including a reduction in bacteria known to produce short chain fatty acids (SCFA) and increased biomarkers of gut permeability and inflammation. Furthermore, we could show that both malnutrition and drug intake are factors associated with microbiome composition in dementia. This study therefore supports the concept of a disrupted gut-brain axis in dementia.

The concept of a disrupted gut-brain axis in dementia has recently emerged. Several animal studies show that induction of dysbiosis by antibiotics, irradiation or germ-free conditions negatively impact on cognitive function and plaque deposition as recently reviewed by Ticinesi et al. [38] Recently, in patients with dementia a reduction in diversity of the microbiome has been described, however data on taxonomic microbiome composition are varying between different studies from different geographical locations [9, 10, 39]. We describe altered beta diversity and distinct taxonomic changes in dementia in a European cohort. Alpha diversity data in dementia so far are conflicting, since a lower alpha diversity has been observed in the study from the USA [9], whereas in the Japanese study a lower alpha diversity was observed in the control group [10] but we found unchanged alpha diversity of the gut microbiome between European dementia patients and healthy age matched controls. Microbial diversity depends on many factors, especially in elderly, where the microbiome is likely to be less stable [40]. Elderly people are more often exposed to microbial community-altering events, such as infections and concomitant antibiotic use, polypharmacy or hospital stays. Therefore, elderly controls may not be healthy in a strict sense and selection of the control cohorts may account for the different findings in different studies. Differences in analysis techniques [41] and also in geographic location [42] may be further factors that impact on diversity and composition of the gut microbiome.

When looking at taxonomic differences, abundance of *Eubacterium rectale*, an uncultured *Lachnospiraceae sp*. and *Lachnospiraceae NK4A136 group* was lower in dementia patients compared to controls. LEfSe also identified the family *Lachospiraceae* with its genus *Lachnospiraceae NK4A136* and several *Lachnospiraceae* species to be associated with health. *Eubacterium rectale* is a well-known butyrate producing bacterium [43] and has already previously been associated with cognitive decline [44]. Members of the *Lachnospiraceae* family have been linked to obesity on the one hand and protection from colon cancer in humans on the other hand, likely due to the association of many species within the group with the production of butyric acid, a SCFA that is important for host epithelial cell growth and integrity [45]. Mild dementia was also associated with another butyate producer – *F. prausnitzii* [46]. Increasing the number of butyrate producing bacteria in dementia may therefore be a promising therapeutic approach, since SCFA such as butyrate are critically involved in microglia maturation and function [2, 3]. Data from animal and human pilot studies support this concept. A dietary intervention with bilberry anthocyanin extract was able to increase *Lachnospiraceae NK4A136 group* abundance and improve gut barrier function in ageing rats [47]. An exploratory pilot study in patients with dementia showed that a multispecies probiotic can increase the abundance of butyrate producing bacterial strains [48]. We also found that the abundance of *C. clostridioforme* and the genus *Eisenbergiella* increased with increasing cognitive impairment. *C. clostridioforme* has mainly been described as a human pathogen [49] but has also been described to be associated with vegetarian diet [50]. The genus *Eisenbergiella* was recently found to be increased in long lived adults [51]. Therefore these findings are difficult to interpret in the context of cognitive dysfunction. The family *Lactobacillaceae* was differentially abundant in different stages of dementia, with the highest abundance in moderate dementia and a lower abundance in mild dementia and severe dementia. LEfSe also revealed the bacterium *L. amylovorus* and the corresponding genus *Lactobacillus*, the family *Lactobacillaceae* and the order *Lactobacillales* to be associated with moderate dementia. This finding is also difficult to interpret, since *Lactobacillus sp.* in general were already more than 100 years ago associated with longevity by the Nobel prize winner Elie Metchnikow in 1907 [52] and several studies using different *Lactobacillus sp.* have been conducted with varying success in neurodegenerative diseases [53]. *Lactobacillus amylovorus* has been described as a novel probiotic strain that is able to reduce ammonia levels and may therefore be associated with cognitive function [54].

Our study also shows that dementia is not only associated with dysbiosis but also associated with markers of increased gut permeability (DAO) and markers of inflammation (sCD14). Ageing itself has been associated with an increase in gut permeability, mucosal inflammation and bacterial translocation – often termed as “inflammageing” [2]. Increased calprotectin levels in stool as a sign of intestinal inflammation have been observed in a pilot study [55]. Another study in dementia showed a decrease in previously elevated zonulin levels after probiotic treatment as a possible hint towards a causal link between dysbiosis and gut permeability in dementia [48]. Although we did not find any differences in stool zonulin and calprotectin levels, we found an increase in DAO levels which has been proven to be a valuable serum biomarker of gut barrier dysfunction [56–59]. Also, Ginko biloba, a commonly used phytotherapeutic drug in dementia, was able to reduce DAO levels in an animal model of alcoholic liver disease, indicating both the validity of DAO as a permeability biomarker and that gut hyperpermeability may be a modifiable and relevant therapeutic target [60]. We furthermore found elevated sCD14 levels in dementia as a marker of endotoxemia and inflammation. Recent in vitro data suggest that the altered stool microbiome composition in dementia directly modulates intestinal epithelial homeostasis via the anti-inflammatory P-glycoprotein pathway [11].

In our cohort, dementia patients, although not different regarding age and gender from our controls, received 3 times more prescription drugs. Although some of these drugs were only prescribed on demand, this finding is still striking. The known consequences of polypharmacy are among others, cognitive impairment, a higher risk of falls and non-compliance, but interventions to reduce polypharmacy are difficult [61, 62]. Drug-microbiome interactions are increasingly recognized. A population based deep sequencing study revealed, that proton pump inhibitors (PPI) were associated with the most profound microbiome changes, followed by statins, antibiotics, laxatives and beta blockers [17]. It has been shown experimentally that not only classic antimicrobials but also many other human-targeted drugs have an extensive impact on human gut bacteria [15]. We recently showed that PPI are one of the main drivers of dysbiosis in liver cirrhosis [63, 64]. We therefore assessed the association of prescription drugs with gut microbiome composition. As expected, effects on overall community structure (alpha and beta diversity) were small. Each drug class was associated with distinct associations throughout different taxonomic levels between users and non-users. PPI use was associated with higher abundance of oral bacteria in the stool and statins and antihypertensive use was associated with an increase in SCFA producing bacteria. Due to the small sample size, the results have to be interpreted with caution and can only serve as pilot data that need to be explored in larger cohorts. Additionally, we assessed the impact of drug intake on markers of gut permeability, bacterial translocation and inflammation. We found that PPI use was associated with increased intestinal inflammation. This has been previously described in the context of other diseases [65–67] and we have recently linked dysbiosis, gut permeability and intestinal inflammation to adverse outcome in patients with liver cirrhosis who use PPI [63]. Antihypertensive use was associated with slightly, but significantly elevated CRP levels, which is most likely due to the underlying disease and not to the drug itself, since arterial hypertension is associated with elevated CRP levels [68] and therefore validates the relevance of our findings although sample size is small.

Malnutrition is common in dementia and nutrition care is an integral part of dementia care [69]. Although all patients in our dementia cohort were treated according to nutritional support standards that include oral nutritional supplements in patients with MNA-SF < 9, MNA-SF and laboratory parameters showed that more than three quarter of the dementia patients in our study were malnourished. It is therefore impossible from this cross-sectional pilot study to distinguish if malnutrition or dementia are the starting point of dysbiosis. This could only be answered by longitudinal studies. In general, malnutrition has been associated with differences in microbiome composition, such as loss of bifidobacteria, however, most studies were performed in malnourished children and not in elderly people [70].

In order to identify the drivers of dysbiosis we performed multivariate analyses with all variables and excluded variables that showed high collinearity to understand the driver of dysbiosis. We found that BMI and statin use were the strongest influencing factors, underpinning the notion that malnutrition and prescription drug use drive microbiome composition in dementia. Also network analysis supports the close association of these factors. However, due to the small sample size, the results of our multivariate analysis have to be interpreted with caution and can be seen as hypothesis generation only. The results need to be confirmed in larger studies.

Our study has some limitations: First, the single center approach and the sample size limit the generalizability of the data. A combination of all studies on gut microbiome in dementia would be desirable, however, due to the lack of standards in sequencing techniques this would not be technically feasible. Second, we could not perform the gold standard analysis of gut permeability – the differential sugar absorption test – because of the cognitive impairment of our patients, who were not able to follow the instructions of the test. We overcame this by using a panel of serum and stool markers that do not require compliance with test instructions for the participants. And third, this study only provides cross-sectional data and can therefore not answer any questions regarding causality or cause-effect relationship between cognitive dysfunction dysbiosis and malnutrition. A longitudinal study is in planning.

## Conclusion

In summary this study provides evidence that structural changes in microbiome composition in dementia are associated with malnutrition and prescription drug use and that biomarkers of gut permeability are increased in dementia. Further studies to move from associations to causality in understanding the gut-brain axis in dementia are necessary. Increasing butyrate producing bacteria and targeting malnutrition seems to be promising therapeutic approaches to treat dementia related dysbiosis. The effect of microbiome modulating strategies on cognitive function needs to be addressed in future studies.

## Supplementary information

**Additional file 1: Figure S1.** Alpha diversity in stool microbiome between dementia patients and controls A: Alpha diversity (Faith PD) in dementia patients and controls B: Alpha diversity (Faith PD) in controls and different stages of cognitive dysfunction C: Alpha diversity (Simpson) between dementia patients a D: Alpha diversity (Simpson) in controls and different stages of cognitive dysfunction. **Table S1.** Drug intake in dementia patients and controls. **Table S2.** Features selected by LEfSe to discriminate between PPI users and non-users. **Table S3.** Features selected by LEfSe to discriminate between statin users and non-users. **Table S4.** Features selected by LEfSe to discriminate between antihypertensive users and non-users. **Table S5.** Features selected by LEfSe to discriminate between NSAID users and non-users. **Table S6.** Features selected by LEfSe to discriminate between thyroid hormone users and non-users. **Table S7.** Features selected by LEfSe to discriminate between malnourished and non-malnourished participants. **Table S8.** Redundancy analysis with explanatory variables of microbiome composition changes.

## Data Availability

Sequence data is publicly available at the NCBI Sequence Read Archive (SRA accession: PRJNA608281). The remaining datasets used and/or analyzed during the current study are available from the corresponding author on reasonable request.

## Abbreviations

AD: Alzheimer’s disease
ANCOM: Analysis of Composition of Microbiomes
BMI: Body mass index
CRP: C reactive protein
DAO: Diamino oxidase
LEfSe: Linear Discriminant analysis Effect Size
LBP: Lipopolysaccharide binding protein
MMSE: Mini Mental State Examination
MNA-SF: Mini Nutritional Assessment Short Form
NSAIDS: Nonsteroidal anti-inflammatory drugs
PPI: Proton pump inhibitors
RDA: Redundancy Analysis
SCFA: Short chain fatty acids
sCD14: Soluble CD14 levels
VIF: Variance inflation factor
