# Supplementary material for: Dysbiosis, gut barrier dysfunction and inflammation in dementia: a pilot study
Source: BMC Geriatr. 2020 Jul 20;20:248. doi: 10.1186/s12877-020-01644-2 (PMC7372911; doi:10.1186/s12877-020-01644-2)
Supplement: Supplementary file 1 — Additional file 1: Figure S1. Alpha diversity in stool microbiome between dementia patients and controls A: Alpha diversity (Faith PD) in dementia patients and controls B: Alpha diversity (Faith PD) in controls and different stages of cognitive dysfunction C: Alpha diversity (Simpson) between dementia patients a D: Alpha diversity (Simpson) in controls and different stages of cognitive dysfunction. Table S1. Drug intake in dementia patients and controls. Table S2. Features selected by LEfSe to discriminate between PPI users and non-users. Table S3. Features selected by LEfSe to discriminate between statin users and non-users. Table S4. Features selected by LEfSe to discriminate between antihypertensive users and non-users. Table S5. Features selected by LEfSe to discriminate between NSAID users and non-users. Table S6. Features selected by LEfSe to discriminate between thyroid hormone users and non-users. Table S7. Features selected by LEfSe to discriminate between malnourished and non-malnourished participants. Table S8. Redundancy analysis with explanatory variables of microbiome composition changes. [file 12877_2020_1644_MOESM1_ESM.docx]

**Supplementary information**

**Dysbiosis, gut barrier dysfunction and inflammation in dementia: A pilot study**

Vanessa Stadlbauer*^1^, Lara Engertsberger*^1^, Irina Komarova^1^, Nicole Feldbacher^1,2^, Bettina Leber^3^, Gerald Pichler^4^, Nicole Fink^4^, Monika Scarpatetti^4^, Walter Schippinger^4^, Reinhold Schmidt^5^, Angela Horvath^1,2^

1 Department of Internal Medicine, Division of Gastroenterology and Hepatology, Medical University of Graz, Graz, Austria

2 Center of Biomarker Research in Medicine (CBmed), Graz, Austria

3 Department of Surgery, Division of Transplantation Surgery, Medical University of Graz, Graz, Austria

4 Department of Internal Medicine and Acute Geriatrics, Geriatric Health Centres Graz, Albert Schweitzer Hospital, Graz, Austria.

5 Clinical Division of Neurogeriatrics, Department of Neurology, Medical University of Graz, Austria.

Supplementary Figure 1


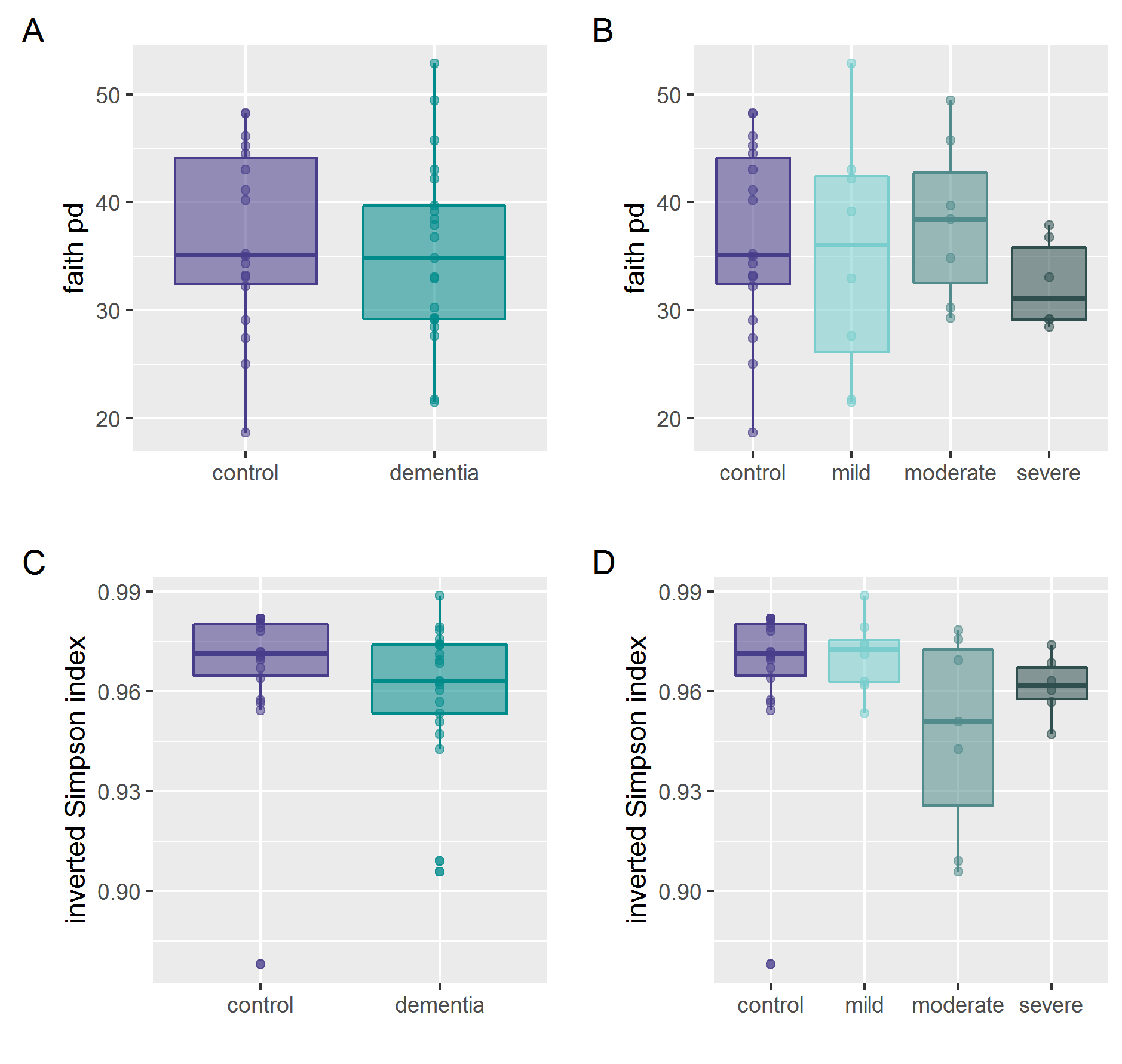


Alpha diversity in stool microbiome between dementia patients and controls A: Alpha diversity (Faith PD) in dementia patients and controls B: Alpha diversity (Faith PD) in controls and different stages of cognitive dysfunction C: Alpha diversity (Simpson) between dementia patients a D: Alpha diversity (Simpson) in controls and different stages of cognitive dysfunction

Supplementary Tables

Table S1: Drug intake in dementia patients and controls

|  | Dementia patients (n=23) | Controls (n=18) | p-value |
| --- | --- | --- | --- |
| Antidepressants (n) | 17 | 1 | <0.001 |
| Laxatives (n) | 11 | 0 | <0.001 |
| Opioides (n) | 11 | 1 | 0.002 |
| Anti-dementia drugs (n) | 14 | 0 | <0.001 |
| Sedatives (n) | 13 | 1 | <0.001 |
| Vitamin D (n) | 17 | 2 | <0.001 |
| Metamizole (n) | 9 | 1 | 0.011 |
| **PPI (n)** | **5** | **8** | **ns** |
| **Antihypertensives (n)** | **13** | **11** | **ns** |
| **Statins (n)** | **2** | **7** | **ns** |
| **NSAIDS (n)** | **7** | **8** | **ns** |
| Paracetamol (n) | 4 | 0 | ns |
| Antidiabetics (n) | 3 | 1 | ns |
| **Thyroid hormones (n)** | **7** | **4** | **ns** |
| Calcium and magnesium supplements (n) | 4 | 2 | ns |
| Anticoagulation (n) | 2 | 4 | ns |
| Phytotherapeutics (n) | 4 | 2 | ns |

PPI proton pump inhibitor, NSAIDS non-steroidal anti-inflammatory drugs, bold print: drug classes selected for further analysis due to distribution and frequency.

Table S2: Features selected by LEfSe to discriminate between PPI users and non-users

|  | **LDA score** | **Associated with** |
| --- | --- | --- |
| **Features** |  |  |
| Bacteroides vulgatus | 3.67 | PPI users |
| *Streptococcus salivarius | 3.55 | PPI users |
|  |  |  |
| **Genera** |  |  |
| Streptococcus | 3.74 | PPI users |
| Barnesiella | 3.68 | PPI users |
| Paraprevotella | 3.54 | PPI users |
| *Holdemanella | 3.64 | non-users |
| Ruminococcaceae NK4A214 group | 3.56 | non-users |
|  |  |  |
| **Families** |  |  |
| *Streptococcaceae | 4.03 | PPI users |
| *Barnesiellaceae | 4.00 | PPI users |
| Elusimicrobiaceae | 4.00 | PPI users |
|  |  |  |
| **Order** |  |  |
| Unclassified | 3.72 | PPI users |

LDA Linear discriminant analysis

*indicates features and genera also found to discriminate between PPI users and non-users

Table S3: Features selected by LEfSe to discriminate between statin users and non-users

|  | **LDA score** | **Associated with** |
| --- | --- | --- |
| **Features** |  |  |
| Erysipelatoclostridium ramosum | 3.52 | non-users |
| Agathobacter sp. | 3.78 | statin users |
| Ruminococcaceae UCG-014 sp. | 3.66 | statin users |
| Massiliprevotella massiliensis | 3.61 | statin users |
| *Faecalibacterium prausnitzii | 3.61 | statin users |
| Lachnospiraceae bacterium MC-35 | 3.60 | statin users |
| Subdoligranulum sp. | 3.55 | statin users |
| Tyzzerella 3 sp. | 3.53 | statin users |
|  |  |  |
| **Genera** |  |  |
| Escherichia-Shigella | 3.76 | non-users |
| *Erysipelatoclostridium | 3.55 | non-users |
| Agathobacter | 3.93 | statin users |
| Prevotella 7 | 3.63 | statin users |
| Coprococcus 2 | 3.55 | statin users |
| Eubacterium eligens group | 3.55 | statin users |
| Lachnospiraceae ND3007 group | 3.55 | statin users |
| Butyrivibrio | 3.53 | statin users |
|  |  |  |
| **Families** |  |  |
| *Enterobacteriaceae | 4.16 | non-users |
| Peptostreptococcaceae | 4.14 | non-users |
| Lachnospiraceae | 4.25 | statin users |
| Ruminococcaceae | 4.16 | statin users |
|  |  |  |
| **Order** |  |  |
| *Enterobacteriales | 4.56 | non-users |
|  |  |  |
| **Classes** |  |  |
| *Coriobacteriia | 3.88 | non-users |
| Actinobacteria | 3.76 | non-users |
|  |  |  |
| **Phyla** |  |  |
| *Firmicutes | 4.45 | statin users |
| *Actinobacteria | 4.39 | non-users |

LDA Linear discriminant analysis

*indicates features and genera also found to discriminate between statin users and non-users

Table S4: Features selected by LEfSe to discriminate between antihypertensive users and non-users

|  | **LDA score** | **Associated with** |
| --- | --- | --- |
| **Feature** |  |  |
| Ruminiclostridium 6 sp. | 3.50 | antihypertensive users |
|  |  |  |
| **Genera** |  |  |
| *Ruminococcaceae NK4A214 group | 3.72 | antihypertensive users |
| Unclassified | 3.53 | antihypertensive users |
| Bacteroides | 4.08 | non-users |
| Blautia | 3.66 | non-users |
| Clostridium innocuum group | 3.63 | non-users |
|  |  |  |
| **Families** |  |  |
| Unclassified | 4.10 | antihypertensive users |
| Prevotellaceae | 4.04 | antihypertensive users |
| Bacteroidaceae | 4.36 | non-users |
| *Lactobacillaceae | 3.81 | non-users |
|  |  |  |
| **Order** |  |  |
| Bacteroidales | 4.64 | non-users |
|  |  |  |
| **Class** |  |  |
| Bacteroidia | 4.63 | non-users |
|  |  |  |
| **Phylum** |  |  |
| Bacteroidetes | 4.71 | non-users |

LDA Linear discriminant analysis

*indicates features and genera also found to discriminate between antihypertensive users and non-users

Table S5: Features selected by LEfSe to discriminate between NSAID users and non-users

|  | **LDA score** | **Associated with** |
| --- | --- | --- |
| **Features** |  |  |
| *Bacteroides sp. | 3.69 | NSAID users |
| Anaerostipes hadrus | 3.52 | NSAID users |
|  |  |  |
| **Genera** |  |  |
| *Anaerostipes | 3.74 | NSAID users |
| Terrisporobacter | 3.65 | NSAID users |
| Intestinimonas | 3.59 | NSAID users |
|  |  |  |
| **Families** |  |  |
| Unclassified | 4.02 | NSAID users |
| Peptococcaceae | 3.74 | NSAID users |
|  |  |  |
| **Order** |  |  |
| Izimaplasmatales | 3.93 | NSAID users |
|  |  |  |
| **Class** |  |  |
| Mollicutes | 3.94 | NSAID users |
|  |  |  |
| **Phylum** |  |  |
| Tenericutes | 4.03 | NSAID users |

NSAIDS non-steroidal anti-inflammatory drugs, LDA Linear discriminant analysis

*indicates features and genera also found to discriminate between NSAIDS users and non-users

Table S6: Features selected by LEfSe to discriminate between thyroid hormone users and non-users

|  | **LDA score** | **Associated with** |
| --- | --- | --- |
| **Features** |  |  |
| *Bacteroides sp. | 3.79 | thyroid hormone users |
| Bacteroides vulgatus | 3.75 | thyroid hormone users |
| Clostridium clostridioforme | 3.60 | thyroid hormone users |
| Ruminococcus 1 sp. | 3.53 | thyroid hormone users |
|  |  |  |
| **Genera** |  |  |
| Blautia | 3.79 | thyroid hormone users |
| Parasutterella | 3.69 | thyroid hormone users |
| *Lactococcus | 3.56 | thyroid hormone users |
| Akkermansia | 3.53 | thyroid hormone users |
|  |  |  |
| **Family** |  |  |
| Akkermansiaceae | 3.68 | thyroid hormone users |
|  |  |  |
| **Orders** |  |  |
| Verrucomicrobiales | 3.73 | thyroid hormone users |
| Unclassified | 3.61 | thyroid hormone users |
|  |  |  |
| **Class** |  |  |
| Verrucomicrobiae | 3.58 | thyroid hormone users |
|  |  |  |
| **Phylum** |  |  |
| Verrucomicrobia | 3.96 | thyroid hormone users |

LDA Linear discriminant analysis

*indicates features and genera also found to discriminate between thyroid hormon users and non-users

Table S7: Features selected by LEfSe to discriminate between malnourished and non-malnourished participants

|  | **LDA score** | **Associated with** |
| --- | --- | --- |
| **Feature** |  |  |
| *Ruminococcaceae UCG-014 sp. | 3.56 | normal |
|  |  |  |
| **Genera** |  |  |
| Eubacterium hallii group | 3.63 | malnutrition |
| Ruminococcaceae UCG014 | 3.77 | normal |
| *Lachnospiraceae NK4A136 group | 3.51 | normal |
|  |  |  |
| **Order** |  |  |
| Unclassified | 3.94 | malnutrition |

LDA Linear discriminant analysis

*indicates features and genera also found to discriminate between dementia and control

Table S8: Redundancy analysis with explanatory variables of microbiome composition changes.

| **Variable** | **Controls&Dementia** | **Severity of dementia** |
| --- | --- | --- |
| BMI | Variance = 32.85  F = 1.26  **P = 0.014** | Variance = 32.85  F = 1.26  **P = 0.009** |
| Albumin | Variance = 42.41  F = 1.64  **P = 0.001** | Variance = 42.41  F = 1.64  **P = 0.001** |
| Total protein | Variance = 32.44  F = 1.24  **P = 0.016** | Variance = 32.44  F = 1.24  **P = 0.01** |
| soluble CD14 | Variance = 36.36  F = 1.4  **P = 0.001** | Variance = 36.36  F = 1.4  **P = 0.001** |
| DAO | Variance = 27.8  F = 1.06  P = 0.196 | Variance = 27.8  F = 1.06  P = 0.201 |
| PPI | Variance = 26.47  F = 1.01  P = 0.417 | Variance = 26.47  F = 1.01  P = 0.398 |
| Statins | Variance = 35.58  F = 1.36  **P = 0.002** | Variance = 35.58  F = 1.36  **P = 0.001** |
| Antihypertensives | Variance = 27.61  F = 1.05  P = 0.158 | Variance = 27.61  F = 1.05  P = 0.205 |
| NSAIDS | Variance = 29  F = 1.1  **P = 0.086** | Variance = 29  F = 1.1  **P = 0.093** |
| Thyroid hormones | Variance = 25.12  F = 0.95  P = 0.721 | Variance = 25.12  F = 0.95  P = 0.711 |
| Zonulin | Variance = 28.03  F = 1.07  P = 0.202 | Variance = 28.03  F = 1.07  P = 0.219 |
| Calprotectin | Variance = 23.29  F = 0.88  P = 0.848 | Variance = 23.29  F = 0.88  P = 0.857 |
| endotoxin | Variance = 20.5  F = 0.77  P = 0.987 | Variance = 20.5  F = 0.77  P = 0.986 |
| LBP | Variance = 25.53  F = 0.97  P = 0.632 | Variance = 25.53  F = 0.97  P = 0.625 |
| Peptidoglycane | Variance = 23.54  F = 0.89  P = 0.868 | Variance = 23.54  F = 0.89  P = 0.866 |
| bacterial DNA | Variance = 24.96  F = 0.95  P = 0.671 | Variance = 24.96  F = 0.95  P = 0.697 |
| MNA-SF | Variance = 36.27  F = 1.39  **P = 0.002** | Variance = 36.27  F = 1.39  **P = 0.001** |
| MMSE | Variance = 44.25  F = 1.71  **P = 0.001** | Variance = 44.25  F = 1.71  **P = 0.001** |
| Clock-drawing test | Variance = 36.26  F = 1.39  **P = 0.005** | Variance = 36.26  F = 1.39  **P = 0.001** |
| Age | Variance = 29.86  F = 1.14  P = 0.062 | Variance = 29.86  F = 1.14  P = 0.075 |
| Sex | Variance = 36.06  F = 1.38  **P = 0.001** | Variance = 36.06  F = 1.38  **P = 0.003** |
| Number of drugs | Variance = 34.81  F = 1.34  **P = 0.004** | Variance = 34.81  F = 1.34  **P = 0.002** |
| CRP | Variance = 24.38  F = 0.92  P = 0.747 | Variance = 24.38  F = 0.92  P = 0.72 |

BMI body mass index, DAO diamino oxidase, PPI proton pump inhibitor, NSAIDS non-steroidal anti-inflammatory drugs, LBP lipopolysaccharide binding protein, MNA-SF Mini Nutritional Assessment Short Form, MMSE mini mental state examination, CRP C reactive protein
